# Identification of p38 MAPK as a novel therapeutic target for Friedreich’s ataxia

**DOI:** 10.1038/s41598-018-23168-x

**Published:** 2018-03-22

**Authors:** M. Grazia Cotticelli, Shujuan Xia, Avinash Kaur, Daniel Lin, Yongping Wang, Eric Ruff, John W. Tobias, Robert B. Wilson

**Affiliations:** 1 Department of Pathology and Laboratory Medicine, Children’s Hospital Philadelphia, Philadelphia, PA USA; 2 The Penn Medicine/CHOP Center of Excellence for Friedreich’s Ataxia Research, Philadelphia, USA; 3 Marian University College of Osteopathic Medicine, Indianapolis, USA; 4 State University of New York Downstate College of Medicine, New York, USA; 5 Perelman School of Medicine at the University of Pennsylvania, Philadelphia, USA

## Abstract

Friedreich ataxia (FRDA) is an autosomal recessive neuro- and cardio-degenerative disorder caused by decreased expression of frataxin, a protein that localizes to mitochondria and is critical for iron-sulfur-cluster (ISC) assembly. There are no proven effective treatments for FRDA. We previously screened a random shRNA library and identified a synthetic shRNA (gFA11) that reverses the growth defect of FRDA cells in culture. We now report that gFA11 decreases cytokine secretion in primary FRDA fibroblasts and reverts other changes associated with cell senescence. The gene-expression profile induced by gFA11 is remarkably similar to the gene-expression profile induced by the p38 MAPK inhibitor SB203580. We found that p38 phosphorylation, indicating activation of the p38 pathway, is higher in FRDA cells than in normal control cells, and that siRNA knockdown of frataxin in normal fibroblasts also increases p38 phosphorylation. Treatment of FRDA cells with p38 inhibitors recapitulates the reversal of the slow-growth phenotype induced by clone gFA11. These data highlight the involvement of the p38 MAPK pathway in the pathogenesis of FRDA and the potential use of p38 inhibitors as a treatment for FRDA.

## Introduction

Friedreich ataxia (FRDA) is an autosomal recessive neuro- and cardio-degenerative disorder characterized by progressive ataxia, areflexia, dysarthria, sensory loss, and hypertrophic cardiomyopathy. (Recent reviews include those by Koeppen and Mazurkiewicz^1^, Collins^2^, and Gomes and Santos)^3^. In the majority of cases, FRDA is caused by a GAA-triplet repeat expansion in the first intron of both alleles of the frataxin gene, *FXN*, resulting in decreased transcription and decreased protein. Frataxin is a mitochondrial protein critical for the assembly of iron-sulfur-clusters (ISCs), which are prosthetic groups important for the function of many proteins, including aconitase and complexes I, II, and III of the mitochondrial electron-transport chain (ETC). Decreased frataxin is associated with mitochondrial iron overload, mitochondrial dysfunction, and increased oxidative stress. Although ISCs are found in many proteins in many biochemical pathways, much less is known about non-mitochondrial ISC proteins affected by decreased frataxin; to what extent dysfunction in these pathways might contribute to the progression of the disease is still largely unknown.

Many ISC proteins are involved in DNA repair, including MutY (Base-Excision Repair), XPD (Nucleotide-Excision Repair), and FancJ (inter-strand-crosslink repair), and increased DNA damage has been measured directly in peripheral blood cells from individuals with FRDA^4^. In the same study, the gene-expression profiles of patient cells were consistent with the presence of genotoxic stress, which correlated with ages of disease onset, frataxin mRNA expression levels, and scores on the International Cooperative Ataxia Rating Scale (ICARS). Based on these data, it was suggested that FRDA cells might decrease their growth rate as a homeostatic response to chronic genotoxic stress, thereby maximizing lifespan. As expected, frataxin knock-down in a neuronal cell line resulted in mitochondrial dysfunction; however, a decreased growth rate was also observed, as well as a phenotype resembling cellular senescence, including positivity for the senescence marker beta-gal^5^. DNA damage, caused by gamma-irradiation or exhaustive replication, is a known inducer of senescence in fibroblasts, a senescence associated with an increase in cytokine secretion. A Senescence Associated Secretion Phenotype (SASP) has been described in fibroblast cell lines; while the SASP is a consistent feature, each cell line also exhibits unique aspects in its secretion profile^6^. Although triggered by DNA damage, the SASP requires, and is sustained by, activation of the stress kinase p38/MAPK14^7^.

We reported previously that primary fibroblasts derived from individuals with FRDA grow more slowly than normal control fibroblasts, particularly in media with galactose or beta-hydroxybutyrate (BHB) in place of glucose^8^, consistent with the known sensitivity of cells with mitochondrial dysfunction to non-glucose-based media^9^. We took advantage of the particularly robust sensitivity of FRDA fibroblasts to BHB-based media to screen a random shRNA library for reversal of the growth defect. Our library, which is completely random at the nucleotide level, allows for unbiased screening and selection for beneficial phenotypes^10^. Our screening in FRDA fibroblasts led to the identification of a random shRNA clone, gFA11, which consistently reverses the growth defect of primary FRDA fibroblasts in both BHB- and glucose-based media^8^. Sequences from gFA11 can be co-immunoprecipitated by an anti-Argonaute antibody, indicating that it likely exerts its effects through canonical RNAi^8^.

We report herein that clone gFA11 also reverts the abnormal morphological changes and the SASP-like cytokine secretion phenotype associated with decreased FXN expression. Through bioinformatic analysis, we identified p38/MAPK14 as a possible mediator of these phenotypes. We show that p38 phosphorylation is increased in patient-derived cells and that inhibition of p38 also reverses the growth defect of primary FRDA fibroblasts. These results highlight the possible use of p38 inhibitors as therapeutic agents for FRDA.

## Results

### Growth phenotype

Clone gFA11 increased the growth of FRDA fibroblasts in BHB-based medium and in glucose-based medium approximately 1.5- and 2-fold, respectively; these differences reached statistical significance in one week^8^. To rule out that gFA11 simply conferred a non-specific growth advantage unrelated to the FRDA phenotype, we tested whether stable expression of gFA11 increased growth in U937 cells (derived from a histiocytic lymphoma), in MCF-10 cells (derived from mammary epithelium), and in apparently healthy primary human fibroblasts. The growth rate of the U937 and MCF-10 cells in BHB-based medium, or DMEM-glucose, was unaffected, even after two weeks in culture; the growth rate in the normal fibroblasts increased slightly in BHB-based medium, but only after two weeks, and the growth in glucose-based medium was unchanged (data not shown). To rule out that the beneficial effects of gFA11 are specific to fibroblasts, rather than to FRDA cells, we stably expressed gFA11 in 15850 cells, an immortalized FRDA lymphocyte line; gFA11 again conferred a growth advantage in glucose-based medium, reaching statistical significance after only one week (data not shown). These results suggest that while gFA11 does, at least partially, reverse the effects of BHB in fibroblasts, there is also a specific effect on FRDA cells. Of note, we have never seen cell immortalization associated with the introduction of gFA11.

**Figure 1:**
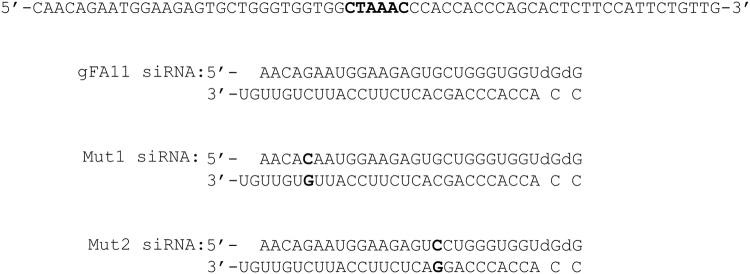
The sequences of gFA11 and variants. The DNA sequence encoding gFA11 is shown on the top line, with the loop sequence in bold. Below this are shown the siRNA sequences of gFA11 and its variants, Mut1 and Mut2, with the variant sequences of Mut1 and Mut2 in bold. Note that in both variants a C/G base-pair is changed to a G/C base-pair, which leaves the pair-bonding dynamics unchanged but would be expected to affect the target profile.

**Figure 2:**
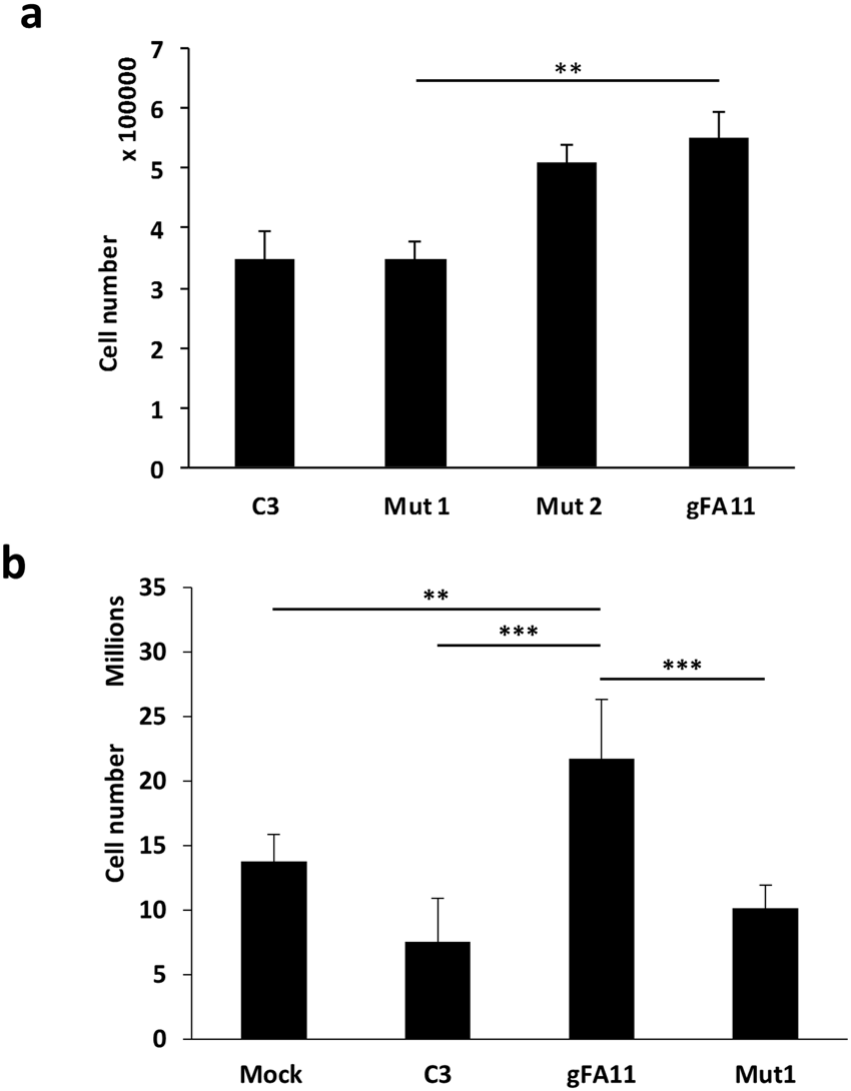
Partial loss of phenotype in seed-sequence variant of gFA11A. (**a**) Primary FRDA fibroblasts GM3816 were transfected with gFA11, Mut1, and Mut2 siRNAs (Fig. 1). The cells were transfected twice over 7 days. Cells were kept in DMEM + 5 mM BHB after the first transfection. **p < 0.01 by ANOVA with Tukey pair-wise comparisons. (**b**) Primary FRDA fibroblasts GM3816 were transfected with gFA11, Mut1, or control (C3) siRNA, or mock transfected, every 3–4 days for a total of four times over 14 days. Cells were kept in DMEM + 5 mM glucose throughout. **p < 0.01 and ***p < 0.005 by ANOVA with Tukey pair-wise comparisons. Error bars represent means ± 1 SD. The averages shown were calculated on three independent replicates and the results are representative of at least two independent experiments.

We previously co-immunoprecipitated processed gFA11 shRNA with Argonaute; sequences derived from both the “sense” and “antisense” strands were recovered, but the “sense” sequence (minus the initial C residue in the predicted shRNA) was much more abundant^8^. These data are consistent with canonical RNAi for the biological activity of gFA11, mediated by the “sense” strand (the first half of the top line in Fig. 1). To further investigate the mechanism of activity of gFA11, we designed two variants of gFA11, each containing a single point mutation (Fig. 1). When these mutated sequences, “Mut1” and “Mut2,” were transfected, as siRNAs, into primary FRDA fibroblasts (GM3816), we found that most of the biological activity of gFA11 in reversing the growth defect in BHB-based medium was lost only with Mut1 (Fig. 2a), which was mutated in the canonical “seed” sequence of the “sense” strand (Fig. 1). Similar results were seen in GM3816 FRDA fibroblasts in glucose-based medium (Fig. 2b) and in the more severely affected GM3665 FRDA fibroblasts (Fig. S1a).

### *In silico* analysis

The availability of Mut1, which differs from clone gFA11 in only a single base but lacks most of gFA11’s biological activity, allowed us to interrogate intracellular pathways affected by gFA11. GM3816 fibroblasts were transfected in triplicate with gFA11 siRNA or Mut1 siRNA four times over two weeks and grown in BHB-based medium. RNA was extracted on day 14 and used for microarray analysis. The microarray results were analyzed using the **D**atabase for **A**nnotation, **V**isualization and **I**ntegrated **D**iscovery (**DAVID**) v6.7 software. The results of Functional Annotation Clustering analysis with the default medium stringency settings are shown in Fig. S2. The annotated references of a statistically significant number of genes were related to secretion in the top two clusters (with Enrichment Scores – ES – of 5.57 and 4.17; Fig. S2). We also performed the analysis using high-stringency parameters and found a significant enrichment for genes involved in cell-cycle regulation (ES of 2.36) and in chemotaxis (ES of 1.96) (data not shown). Strikingly, cytokines and cytokine receptors were at the top of the list of the 301 genes used for the analysis.

We also used our microarray results to perform Ingenuity Pathway Analysis (IPA). This analysis identified the chemical compound SB203580, a known inhibitor of p38 MAP kinase, as an “upstream regulator” (z-score = +3.14). An upstream regulator is defined as a protein, a transcription factor, or a chemical substance that when activated (positive z-score) or inhibited (negative z-score) induces a gene-expression pattern similar to that observed in the microarray data. Taken together, these results suggest a role for p38 MAP kinase as a mediator of the biological activity of gFA11.

### Secretion phenotype

**Figure 3:**
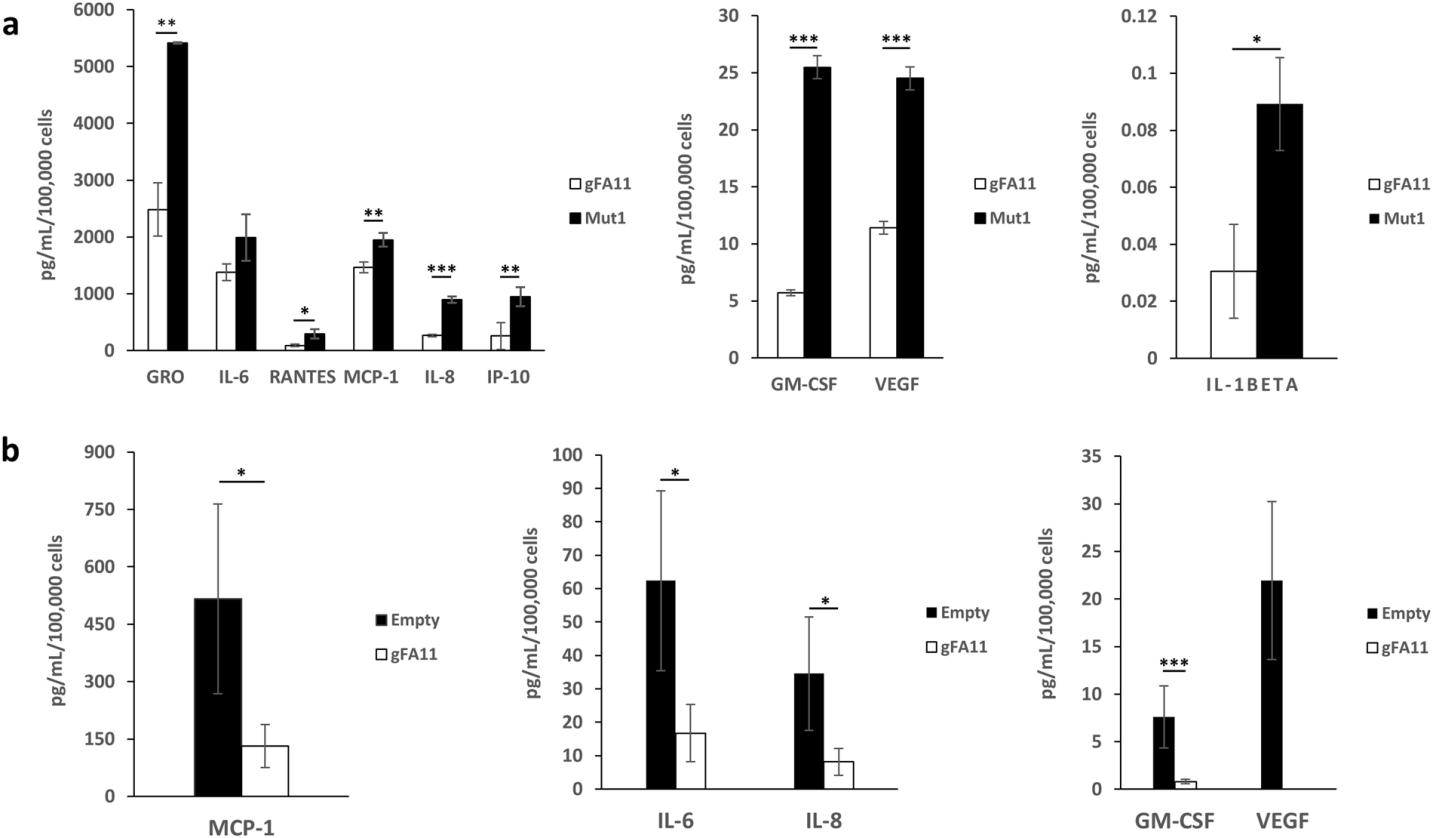
Alterations in cytokine secretion induced by gFA11. (**a**) Primary FRDA GM3816 fibroblasts were transfected in triplicate with gFA11 siRNA or Mut1 siRNA four times over twelve days. After the first transfection, the cells were grown in DMEM plus 5 mM BHB. At day 12, after the fourth transfection, cells were incubated with DMEM with BHB but without FBS. The following day, the medium was collected, concentrated, and analyzed by Luminex assay. Cytokine concentrations were normalized by cell numbers. (**b**) Primary FRDA GM3816 fibroblasts were infected with a gFA11-encoding vector or empty vector (E). Infected cells were selected with puromycin and expanded in glucose-based medium. Cells were then seeded at low density and kept in medium without FBS for 24 hours before collecting, concentrating, and analyzing the medium. *p < 0.05; **p < 0.01; ***p < 0.005 by Student’s t test. Error bars represent means ± 1 SD. The averages shown were calculated on three (**a**) or four (**b**) independent replicates and the results are representative of two independent experiments.

To verify the microarray results, we determined whether gFA11 affects cytokine secretion in primary FRDA fibroblasts. We transfected GM3816 FRDA fibroblasts with gFA11 siRNA or Mut1 siRNA four times over two weeks and cultured the cells in BHB-based medium. After the fourth transfection, cells were switched to DMEM without FBS for 24 h. The conditioned medium (CM) was then concentrated and the concentrations of 13 cytokines in the medium were measured by Luminex assay. The concentrations of eight cytokines – GRO, RANTES, MCP-1, IL-8, IP-10 GM-CSF, VEGF, and IL-1beta – were significantly decreased in cells transfected with gFA11 (p-values < 0.05) compared to cells transfected with Mut1 (Fig. 3a). These data are in agreement with the microarray data, with the exception of GM-CSF (q-value from microarray: 65). Also in agreement with the microarray data, TNF alpha, TNF beta, and IL-12 concentrations were not significantly different between the two cell populations (p-values = 0.2, 0.45, and 0.5, respectively). Finally, the concentration of IL-6 (with q value of 2 from the microarray) was consistently lower in the medium of cells transfected with gFA11, but this did not reach statistical significance (two-sided p-value = 0.08). IL-4 was undetectable in the medium.

To confirm these data, we repeated the experiment with three differences: (1) to rule out effects of siRNA over-treatment, we stably expressed gFA11 shRNA in cells, (2) we used cells into which an empty vector was introduced as a control and, (3) we kept the cells in glucose-based medium throughout. The concentrations of GRO, MCP-1, IL-8, IL-6, IP-10, GM-CSF, and VEGF were all decreased in cells expressing gFA11 (Fig. 3b). VEGF was quantifiable in the control (Mut1-expressing) samples but undetectable in cells expressing gFA11. IP-10 and GRO were below the detection threshold in all samples.

### Morphology and cell cycle analysis

**Figure 4:**
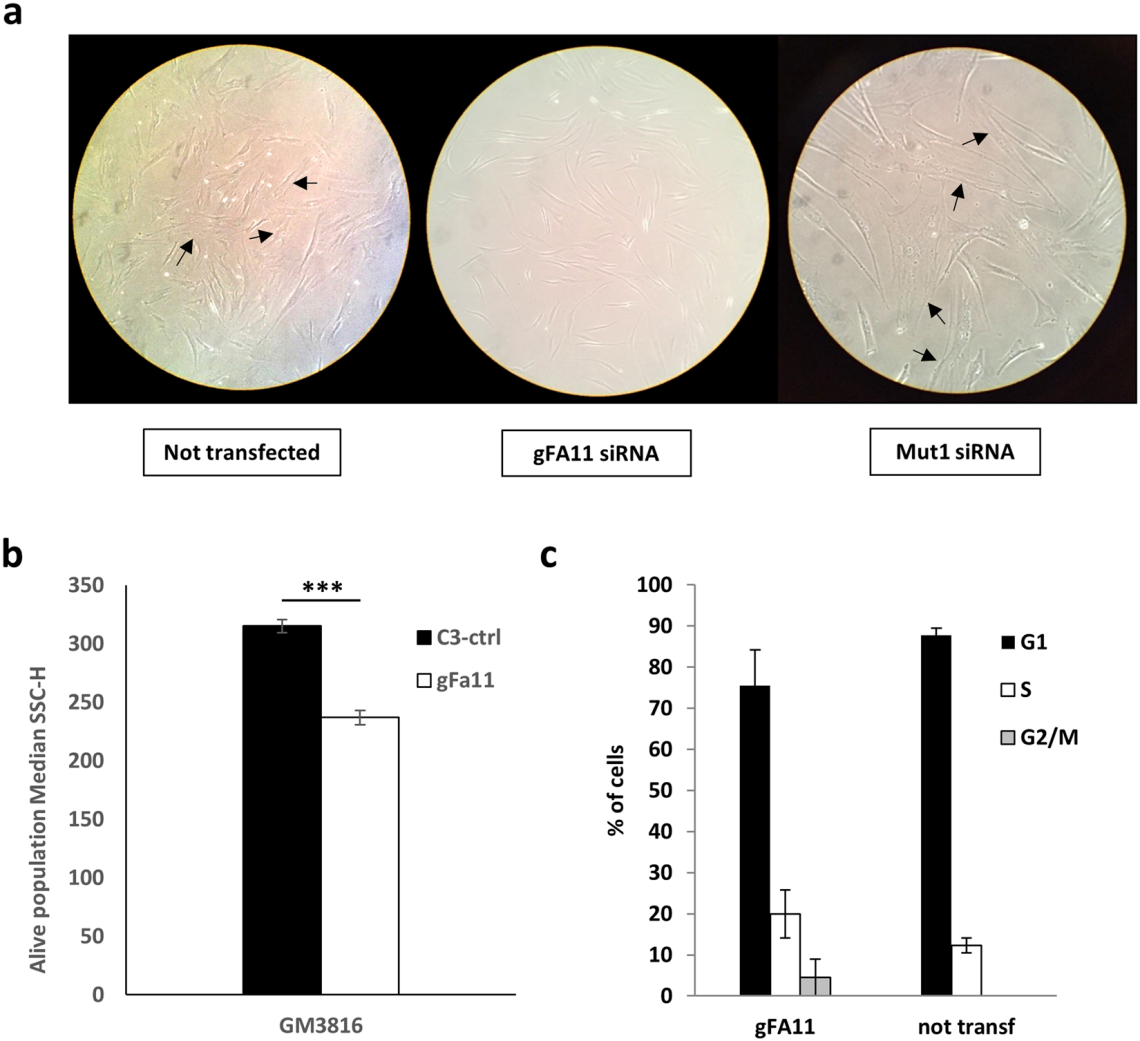
Effects of gFA11 on cell cycle and morphology. (**a**) Morphology of primary FRDA GM3816 fibroblasts transfected with gFA11 siRNA or Mut1 siRNA, or not transfected. Arrows indicate senescent-appearing cells. (**b**) Flow-cytometric side scattering (SSC) of GM3816 and GM3665B cells infected with a gFA11-encoding or control-encoding (C3) vector. ***p < 0.005 by Student’s t test. (**c**) Cell-cycle analysis of GM3816 cells, untransfected or transfected 4 times with gFA11 siRNA over two weeks. Error bars represent means ± 1 SD.

During the course of these experiments, we noticed that the morphology of cells transfected with clone gFA11 was different from that of cells transfected with the clone Mut1 and markedly different from that of untransfected cells (Fig. 4a). Qualitatively, cultures transfected with gFA11 siRNA, or stably expressing gFA11, appeared healthier and had fewer enlarged, granular, senescent-appearing cells. These differences were quantified using side-scatter parameters from flow cytometry plots of GFP-positive GM3816 FRDA fibroblasts stably expressing gFA11 versus a random clone (Fig. 4b). Untransfected GM3816 FRDA fibroblasts, and GM3816 FRDA fibroblasts transfected with gFA11 siRNA, were compared using cell-cycle analysis; untransfected cells in G2/M were undetectable, whereas gFA11-transfected cells in G2/M were quantifiable (Fig. 4c). Similar results were seen with GM3665B FRDA fibroblasts (Fig. S1c).

### Activation of the p38 pathway in FRDA fibroblasts

**Figure 5:**
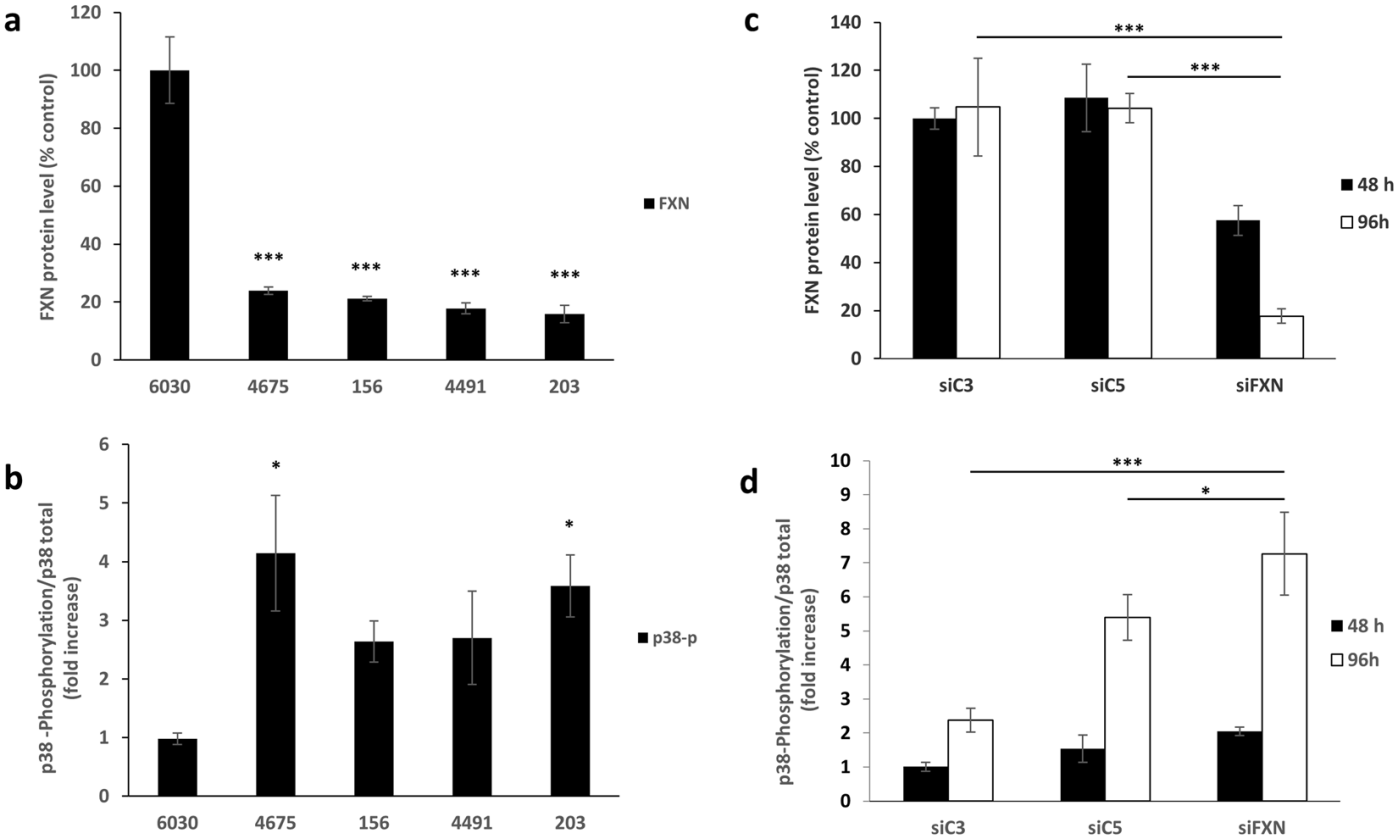
Activation of p38 MAP kinase in FRDA cells. (**a**) Frataxin protein levels (by ELISA) in normal control fibroblasts (6030) and primary FRDA fibroblasts (4675, 156, 4491, 203). ***p < 0.005 by ANOVA with Tukey pair-wise comparisons. (The aggregate p value comparing the normal control to the FRDA cells combined was less than 1 × 10^-8^). (**b**) p38 phosphorylation in the same cells as in (**a**) *p < 0.05 by ANOVA with Tukey pair-wise comparisons. (156 and 4491 cells grew extremely slowly and only two replicates were possible; the aggregate p value comparing the normal control to the FRDA cells combined was 0.008). (**c**) Frataxin protein levels (by ELISA) in normal control fibroblasts (6030) transfected with a random siRNA, C3 (siC3), a known toxic siRNA, C5 (siC5), or an siRNA to frataxin (siFXN) after one transfection (left bar) or two transfections (right bar). The decrease with siFXN was associated with p < 0.005 in both cases by Student’s t test. (**d**) p38 phosphorylation in the same cells as in (**c**) *p < 0.05; ***p < 0.005 by Student’s t test. Error bars represent means ± 1 SD. The averages shown were calculated on three independent replicates and the results are representative of at least two independent experiments.

**Figure 6:**
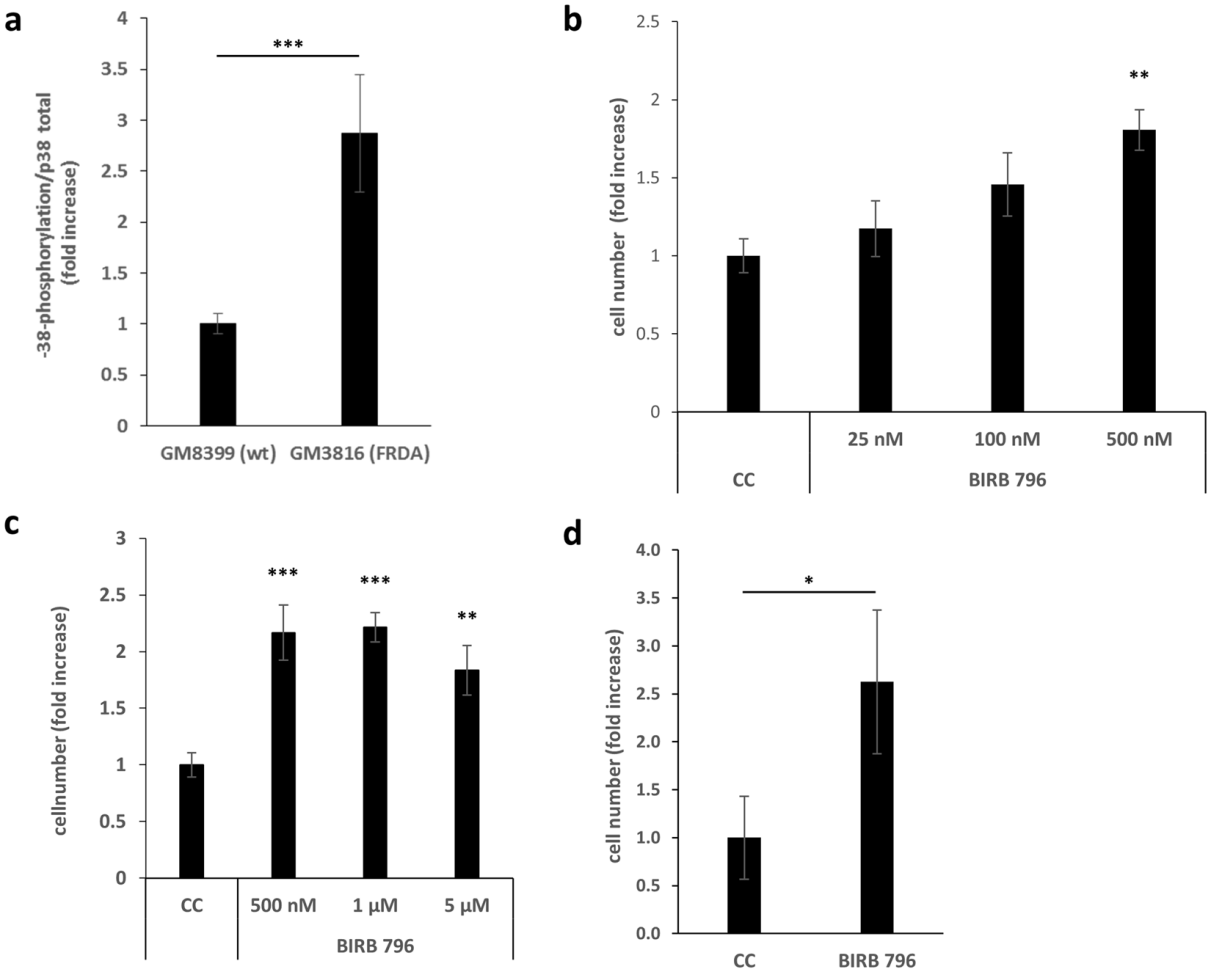
p38 inhibitors increase the growth of FRDA cells in a dose-dependent manner. (**a**) p38 phosphorylation status in control GM8399 fibroblasts and FRDA GM3816 fibroblasts. ***p < 0.005 by Student’s t test. (**b**) Primary FRDA GM3816 fibroblasts treated every 48 h with the p38 inhibitor BIRB796 at the concentrations indicated. Cells were counted at day 14. **p < 0.01 relative to carrier control (CC) by ANOVA with Tukey pair-wise comparisons. (100 nM reached a p value of 0.051 relative to carrier control.) Error bars represent means ± 1 SD. (**c**) Primary FRDA GM3816 fibroblasts treated every 48 h with the p38 inhibitor BIRB796, at the concentrations indicated. Cells were counted at day 7. **(d)** Primary DL156 primary FRDA fibroblasts were treated every 48 h with the p38 inhibitor BIRB796, at a concentration of 500 nM. *p < 0.05 relative to carrier control (CC) by Student’s t test. **p < 0.01; ***p < 0.005; relative to carrier control (CC) by ANOVA with Tukey pair-wise comparisons. Error bars represent means ± 1 SD. In all experiments, the averages shown were calculated on three independent replicates and the results are representative of at least two independent experiments.

The Senescence Associated Secretion Phenotype (SASP) in senescent fibroblasts is sustained through the activation of MAPK14/p38^7^. From Ingenuity Pathway Analysis, the gene-expression profile of cells transfected with gFA11 siRNA was consistent with decreased activation of the p38 pathway. We measured p38 phosphorylation, a marker of activation of the p38 pathway, in primary FRDA fibroblasts and control, normal fibroblasts; in the FRDA cells, in which frataxin levels were ~5–20% of those seen in control cells (Fig. 5a), p38 phosphorylation was more than two-fold higher (Fig. 5b). We then tested whether a decrease in frataxin expression level was sufficient to activate the p38 pathway. Using siRNA directed against frataxin, we knocked down frataxin in normal control fibroblasts, achieving more than 99% knockdown of frataxin mRNA after 48 hours (data not shown). By ELISA, frataxin protein levels decreased to 40% at 48 h after one transfection and decreased to 20% of normal at 96 h after two transfections (Fig. 5c); concomitant increases in p38 phosphorylation were observed, reaching two-fold at 48 h and seven-fold at 96 h (Fig. 5d). Knocking down frataxin was also sufficient to increase IL-6 concentration in the cell-culture medium after one transfection (data not shown). The cells eventually lost viability after several days, presumably due to extremely low levels of frataxin protein, which has a finite half-life, and/or the rapidity of the frataxin decline. To rule out that the observed increase in p38 phosphorylation was simply due to incipient cell death, we included as a further control a toxic siRNA (C5) that we had previously shown to cause cell death over a similar time-frame; although C5 did activate the p38 pathway, the level of p38 phosphorylation in the cells in which frataxin was knocked down was still significantly higher (p < 0.05; Fig. 5d). Moreover, treatment of the cells with p38 inhibitors after each transfection with the frataxin siRNA did not rescue the cells from cell death (data not shown), suggesting that activation of the p38 pathway was not contributing directly to the death signal. Finally, we tested whether treatment with p38 inhibitors could reverse the growth defect of primary, commercially available FRDA fibroblasts (GM3816), in which p38 phosphorylation was approximately three-fold higher than in normal control cells (Fig. 6a). We administered the p38 inhibitor BIRB796 every 48 h and observed a dose-responsive increase in growth after two weeks (Fig. 6b); we tested higher concentrations and found that the growth increase associated with BIRB796 reached statistical significance in only one week (Fig. 6c). Similarly, BIRB796 increased the growth of DL156 primary FRDA fibroblasts (Fig. 6d). Importantly, the growth increase was never more than two-fold, thus matching, but not exceeding, the growth rate of normal control fibroblasts.

**Figure 7:**
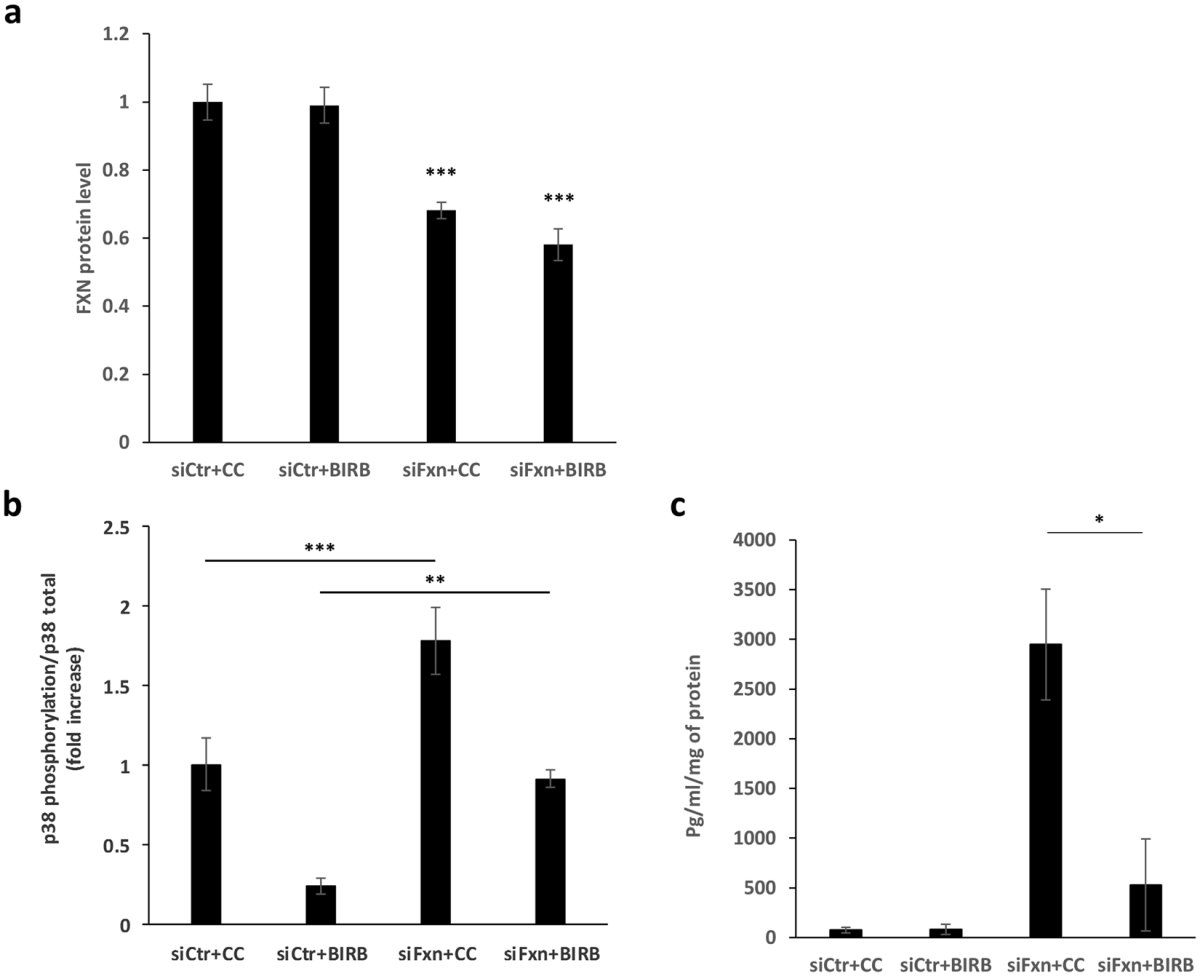
p38 phosphorylation is not sustained by cytokines. Primary apparently healthy fibroblasts GM8399 were transfected with FXN siRNA or a random control clone. Two hours after transfection, cells were incubated with DMEM without FBS in presence of 500 nM BIRB796 or carrier control. The following day, (**a**) frataxin protein levels, (**b**) p38 phosphorylation levels, and (**c**) IL-6 levels were measured by ELISA. **p < 0.01; ***p < 0.005 by ANOVA with Tukey pair-wise comparisons. Error bars represent means ± 1 SD. In all experiments, the averages shown were calculated on three independent replicates and the results are representative of at least two independent experiments.

P38 phosphorylation is associated with cytokine secretion in senescent fibroblasts^7^; conversely, cytokines can induce p38 phosphorylation, particularly in inflammatory diseases^11^. In normal primary fibroblasts in which frataxin was knocked down rapidly to approximately 60% of normal (Fig. 7a), BIRB796 decreased the moderately elevated p38 phosphorylation approximately 50%, to essentially normal levels (Fig. 7b), and decreased elevated IL-6 secretion approximately 80% (Fig. 7c). Consistent with findings from other investigators^12,13^, we found that treatment of normal control cells with BIRB796 also decreased p38 phosphorylation (Fig. 7b); consistent with our results described above, the knockdown of frataxin in the absence of drug was associated with a 1.7-fold increase in phosphorylated p38 (Fig. 7b). Knocking down frataxin resulted in a 40-fold increase in IL-6 secretion (Fig. 7c, first and third bars; p < 0.002 by ANOVA with Tukey pair-wise comparisons). In the presence of drug, the knockdown of frataxin was associated with a 4-fold increase in phosphorylated p38 (Fig. 7b), but the change in IL-6 secretion under these conditions did not reach statistical significance (Fig. 7c, second and fourth bars). Primary GM3816 FRDA fibroblasts exposed to low-cytokine medium from FRDA cells expressing gFA11 (Fig. 3, black bars), or to high-cytokine medium from control FRDA cells expressing Mut1 (Fig. 3, white bars), showed no difference in growth (data not shown). In addition, expression of gFA11 in primary GM3816 FRDA fibroblasts had no effect on p38 phosphorylation (Fig. S3). These data suggest that gFA11 acts downstream of p38, and that p38 phosphorylation in frataxin-deficient cells is not sustained by cytokines.

## Discussion

We identified p38 inhibitors as possible therapeutic agents for Friedreich ataxia through the study of a synthetic shRNA clone that rescues the growth phenotype of FRDA fibroblasts^8^ and reverses hallmarks of cellular senescence, including cell-cycle distribution and cytokine secretion. The Senescence Associated Secretion Phenotype develops under conditions of genotoxic stress, in which cumulative DNA damage induces cell-cycle arrest and, through p38 activation, triggers the secretion of cytokines, particularly IL-6 and IL-8. A chronic, elevated level of DNA damage has been demonstrated in the lymphocytes of FRDA patients^4^. Herein we have shown a higher level both of p38 phosphorylation and cytokine secretion in primary fibroblasts derived from individuals with FRDA.

Whether due to an increase in ROS or due to a deficit in iron-sulfur-cluster-containing DNA repair enzymes (or both), FRDA cells accumulate DNA damage and respond by progressively slowing down the cell cycle (Haugen *et al*.^4^, and references therein) and developing a secretory phenotype through p38 activation. We hypothesize that the damage and corresponding pathway activation are low-level and chronic, allowing the cells to adapt and gradually assume the morphology and behavior of senescent cells while continuing to replicate. Because they are continuing to replicate, albeit more slowly, their state might better be defined as “pseudo-senescent.” Consistent with this hypothesis, FRDA fibroblasts in G2/M are undetectable in culture, a result similar to that seen with neuronal cells in which frataxin was knocked down^5^. Alternatively, the secretion phenotype we observe might derive from a small percentage of cells constantly entering senescence, which seems unlikely given that we worked with small numbers of early-passage cells and generally kept them sub-confluent. In this alternative hypothesis, gFA11 would act to delay entry of a sub-set of FRDA fibroblasts into senescence without necessarily affecting the majority of the cells.

The existence of a secretion phenotype in FRDA fibroblasts introduces a new layer of complexity in our understanding of the disorder as it supports the possibility that non-cell-autonomous effects contribute to the pathophysiology. Mitochondrial dysfunction per se can also induce a type of senescence – Mitochondrial Dysfunction-Associated Senescence or MiDAS – associated with a distinct secretion phenotype^14^. Decreased levels of the mitochondrial sirtuins Sirt3 and Sirt5 induce senescence and a secretory phenotype that lacks IL-6, IL-8 and VEGF^14^. MiDAS is driven by a decreased NAD+/NADH ratio, which results in p53 activation through AMPK phosporylation^14^. Intriguingly, in cardiomyocytes from mouse models of FRDA, Sirt3 activity is decreased secondary to an increased NAD+/NADH ratio^15^. The secretion phenotype of FRDA cells might well involve some combination of these two pathways and therefore might be associated with an unusual combination of cytokines. The cytokine secretion phenotype we observed in FRDA cells was augmented in medium containing beta-hydroxybutyrate, without qualitative changes, at least regarding the cytokines we measured; thus gFA11 and beta-hydroxybutyrate are potentially two important tools to study this phenotype.

The physical targets of gFA11 are under investigation but remain, as yet, unknown. Given that cytokine secretion is regulated post-transcriptionally^16^, we explored the possibility that gFA11 could primarily down-regulate cytokine secretion, which could in turn alter p38 phosphorylation. However, p38 phosphorylation was unchanged in the presence of gFA11 (suggesting that gFA11 acts downstream of p38) and FRDA cells exposed to low- or high-cytokine conditioned medium showed no difference in growth, suggesting that p38 phosphorylation in frataxin-deficient cells is not sustained by cytokines. That cytokine secretion seems to have little or no effect on cultured fibroblasts is in agreement with the findings of Coppe *et al*. that the effects of the SASP are most evident on co-cultured epithelial cells^17^. In the context of FRDA, the effects of a secretory phenotype are yet to be explored.

While gFA11 is highly efficacious, at least in our cell-culture models, the well-known problems associated with the delivery of small RNAs *in vivo* preclude clinical application any time soon. Through bioinformatic analyses of gFA11, we identified p38 inhibitors as inducers of a strikingly similar expression profile and showed that BIRB796, a well-characterized p38 inhibitor, also reverses the growth defect of FRDA fibroblasts. Whereas inhibition of p38-target phosphorylation is generally measured after administration of a single micromolar dose^18^, the effect on cell growth described herein was evident at nanomolar concentrations administered every 48 hours. That low doses, administered to FRDA cells at 48-hour intervals, are efficacious is consistent with the hypothesis that they are counteracting chronic, low-level p38 activation, such as we measure in patient-derived fibroblasts.

Tian *et al*. identified ISCU – a key component of the mitochondrial iron-sulfur-cluster assembly complex – as a downstream target of MAPKAPK2/MK2 and showed that in Drosophila, as well as in mammalian cells, phosphorylation of ISCU results in lower complex I activity^19^. As MK2 is a target of p38 kinases, these data establish a link between the DNA damage response pathway and iron-sulfur-cluster assembly. Modulation of ISC assembly though ISCU phosphorylation is likely maladaptive in the context of FRDA, in which ISC assembly is already compromised due to low frataxin, and may thereby contribute to the phenotype. Strikingly, mutations in the yeast ISCU homolog, Isu1p, can bypass the need for yeast frataxin^20,21^.

One limitation of the present study is that the causal relationship between p38 phosphorylation and cytokine secretion in the context of low frataxin remains to be determined. Knockdown of frataxin in the absence of p38 inhibitors increased both p38 phosphorylation and IL-6 secretion, whereas knockdown of frataxin in the presence of p38 inhibitor increased p38 phosphorylation but not IL-6 secretion. Note, however, that in the latter case, the increase in p38 phosphorylation was from sub-normal back to normal; if p38 phosphorylation per se causes IL-6 secretion in primary fibroblasts with low frataxin, then these results suggest that a certain threshold of p38 phosphorylation needs to be reached. Another limitation of the present study is the fact that, although primary FRDA fibroblasts exhibit many of the hallmarks of FRDA pathophysiology, including mitochondrial iron overload, mitochondrial dysfunction, and increased sensitivity to oxidative stress^22–24^, fibroblasts are not an affected cell type in FRDA *in vivo*. Likewise, we cannot rule out that the phenotypes we have reported herein, including p38 activation and the SASP, are restricted to cultured fibroblasts. Nevertheless, the previous findings of Haugen *et al*. regarding DNA damage in FRDA^4^, the link between the DNA damage response pathway and the ISC assembly machinery through activation of the p38 pathway^19^, and the close correlations between these previous studies and the findings reported herein, are sufficiently compelling that we have initiated the testing of p38 inhibitors in mouse models of FRDA.

The identification of gFA11, a highly efficacious shRNA with no discernable toxicity, validates our phenotypic screening approach to discover viable shRNA therapeutic candidates. With the addition of microarray analyses and bioinformatic pattern-matching, a single screen with our random, shRNA-expressing library potentially yields: small-RNA therapeutic candidates; conventional chemical-compound therapeutic candidates; drug-target candidates; and elucidation of disease mechanisms, which may inform additional therapeutic initiatives.

## Methods

### Cell lines

We obtained primary FRDA fibroblasts, GM3816 and GM3665B; age- and sex-matched apparently healthy control fibroblasts, GM8400 and GM8399; and FRDA lymphocytes, 15850, from Coriell (Coriell Institute for Medical Research, Camden, NJ). The FRDA fibroblasts have homozygous GAA repeat expansions in the first intron of the *FRDA* gene: GM3816 cells have moderate GAA repeat expansions of 223 and 490 repeats, while GM3665B cells have larger GAA repeat expansions of 790 and 1357 repeats. (GM3665B cells grow very slowly and were generally used to confirm results obtained with GM3816 cells, particularly in experiments that did not require a large number of cells.) Primary cells derived from the practice of Dr. David Lynch at the Children’s Hospital of Philadelphia were: apparently healthy fibroblasts (DL6030), FRDA fibroblasts with moderate GAA repeat expansions of 600 and 1200 repeats (DL4675) and with GAA repeat expansions of 500 and 570 repeats (DL156), FRDA fibroblasts with a GAA repeat expansion of 762 repeats and a G130V disease-associated missense mutation (DL4491), FRDA fibroblasts with homozygous GAA repeat expansions of 901 repeats (DL203). Subjects were recruited using the Friedreich’s Ataxia Research Alliance database, by listing on Clinicaltrials.gov (NCT01965327), and through the practice of the Principal Investigator (PI). Declaration of Helsinki protocols were followed and all subjects provided written informed consent prior to participation. The Children’s Hospital of Philadelphia Institutional Review Board approved the study. Subjects were provided with a modest stipend for each visit.

A glucose-free formulation of Dulbecco’s Modified Eagle’s Medium (DMEM) was used for cell culture (Life Technology (11966-025)). To this base medium either glucose or DL-Beta-hydroxy-butyrate (Acros Organic-cat. 215011000) was added at 5 mM final concentration. During cell expansion the medium used was low-glucose DMEM (Life Technology 11885-076). All media were supplemented with 10% fetal bovine serum (Hyclone) and 1% penicillin/streptomycin.

### Cell Growth

Cell growth was quantified by counting cells using the Countess^TM^ Counter (Life Technologies). Pictures were taken using an EVOS microscope. p38 inhibitors were from Selleckchem.

### Cell cycle analysis

Cells were washed, fixed with 70% ethanol and stained with Propidium Iodide at 20 ug/ml for 20 min. The samples were analyzed on the FACSCalibur (FL2 channel, linear mode, FL2 area vs. FL2 width to plot). The analysis was performed with MOD-Fit LT software version 4.1.

### shRNA plasmids and siRNA

Sequences of the shRNA gFA11 and its variants, Mut1 and Mut2, are shown in Fig. 1. DNA and RNA oligonucleotides were from IDT DNA. siRNA against frataxin was from Origene. The gFA11-, Mut1-, and Mut2-expressing sequences were each cloned into the GFP-expressing vector, pSIREN, or in a puromycin-resistant version of the same vector (Clontech) to allow selection either by GFP sorting or by growth in puromycin-supplemented medium (1 ug/ml). To infect human cells, the single clones were packaged using amphitropic retroviral packaging plasmids. Double stranded siRNAs were transfected using the RNAi-max reagent (Life Technology) at a final concentration of 10 nM as per the manufacturer’s instructions. Cells were transfected every 3–4 days up to 14 days^8^. Clones C3 and C5 are random clones from the parent random shRNA-expressing library; C3 has essentially no effect on cells, and was used as a negative control, whereas C5 is toxic to cells, and was used as a cell-death control.

### ELISA

Quantification of frataxin, total and phosphorylated p38, and IL-6 was performed by ELISA using the corresponding Abcam kits as per the manufacturer’s instructions.

### Luminex analysis

Cytokine secretion was measured as described in Coppe *et al*.^17^. GM3816 cells at passage 17 were transfected with gFA11 siRNA or Mut1 siRNA every 3–4 days over twelve days. After the first transfection, cells were kept in BHB-based medium at all times. After the fourth transfection, cells were allowed to recover for 1 h, then medium without FBS was added. GM3816 cells were infected at passage 14, selected, and expanded for at least six weeks. Infected cells were seeded at low density and medium without FBS was added 48 hours later. To harvest cytokines, cells were kept in 4 ml medium without FBS, but containing either BHB or glucose for 24 h. The medium was concentrated using Amicon® Ultra-4 Centrifugal Filter Units (3000 kDa cut-off) and analyzed by Luminex assay using the Human Cytokine MAGNETIC Kit (HCYTOMAG-60K-07.EMD Millipore) at the Human Immunology Core facility of the University of Pennsylvania.

### Microarray analysis

GM3816 fibroblasts were transfected in triplicate with clone gFA11 or clone Mut1 four times over two weeks and grown in BHB-based medium after the first transfection. RNA was extracted at day 14 by using the Qiagen mRNAeasy kit. RNA quality control, reverse transcription, and hybridization to the Human Gene 2.0 ST GeneChips (Affymetrix Inc., Santa Clara CA) were performed by the University of Pennsylvania Molecular Profiling Facility. To quantify expression levels of targeted genes, RMA normalization was applied using Partek Genomics Suite v6.6, yielding log2-transformed normalized intensities. Genes were analyzed for differential expression using Significance Analysis of Microarrays (SAM; samr v2.0)^25^. The gene list was then sorted by q values, a statistical parameter that takes into account the magnitude of the differences, the precision of the data, and correction for multiple comparisons. The q value calculated by SAM represents false discovery rate on a scale of 0–100.

The 301 genes with a q value of zero were analyzed using the **D**atabase for **A**nnotation, **V**isualization and **I**ntegrated **D**iscovery (**DAVID**) v6.7. Functional annotation analysis finds the most overrepresented biological terms associated with a given gene list; the annotation coverage includes more than 40 categories, including GO, KEGG, and BioCarta pathways. An enrichment score (ES) >1.3 is considered significant. Genes that differed in expression >1.5 fold *and* were associated with a q value ≤10 were also analyzed through the use of Ingenuity Pathway Analysis (Ingenuity® Systems, www.ingenuity.com) through the UPenn Molecular Profiling Facility. Microarray data are available in the ArrayExpress database (www.ebi.ac.uk/arrayexpress) under accession number E-MTAB-4699.

### Statistics

For simple comparisons of two means, p values were derived from Student’s t test and are two-sided. Analysis of variance (ANOVA) was performed using R (version 3.4.1), with post-hoc analysis by the Tukey method for pair-wise comparisons. “Independent replicates” within an experiment refers to cells that were plated independently, cultured independently, treated independently (transfected, infected, combined with drug or carrier control), and analyzed independently. Where indicated, we repeated experiments in their entirety to confirm results. Because primary cells change with passage number, and because infection and transfection efficiencies differ from one experiment to the next, we generally did not combine data from confirmatory independent experiments.

### Data availability

The datasets generated during and/or analyzed during the current study are available from the corresponding author on reasonable request.

## Electronic supplementary material

Supplemental Figures
