# Supplementary material for: Identification of p38 MAPK as a novel therapeutic target for Friedreich’s ataxia
Source: Sci Rep. 2018 Mar 22;8:5007. doi: 10.1038/s41598-018-23168-x (PMC5864720; doi:10.1038/s41598-018-23168-x)
Supplement: Supplementary file 1 — Supplemental Figures [file 41598_2018_23168_MOESM1_ESM.pdf]

SUPPLEMENTARY MATERIAL

Identification of p38 MAPK as a novel therapeutic target for Friedreich's ataxia

M. Grazia Cotticelli[1], Shujuan Xia, Avinash Kaur, Daniel Lin, Yongping Wang, Eric Ruff, John W. Tobias, and Robert B. Wilson

Fig. S1

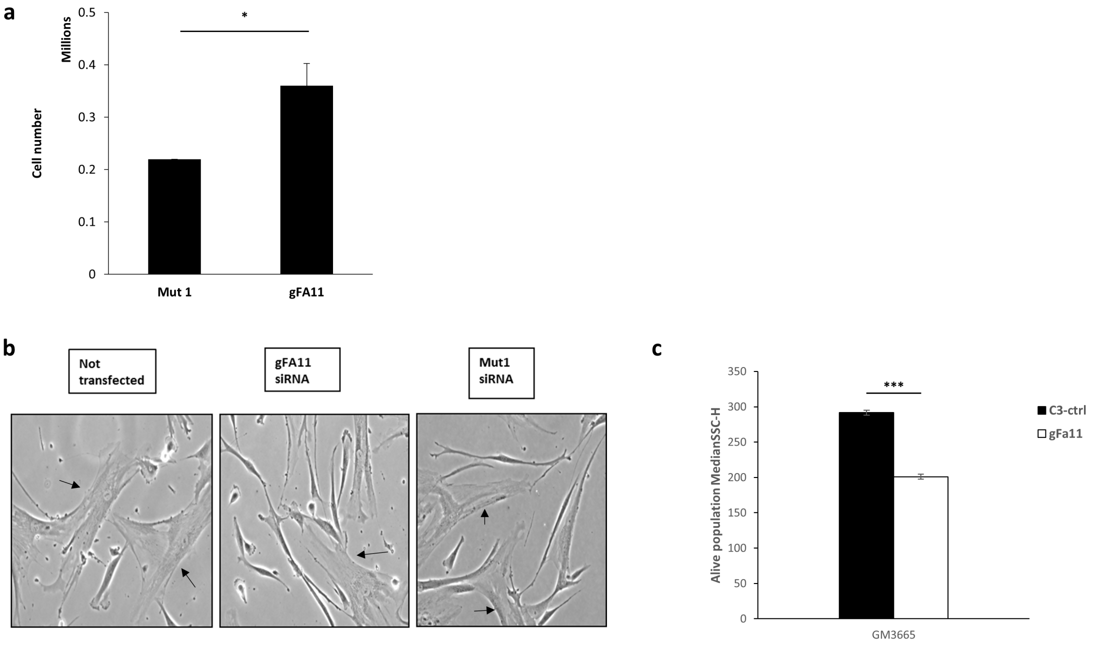

Figure S1. gFA11A rescues the growth and morphology phenotype in GM3665B (**a**). Primary FRDA fibroblasts GM3665B were transfected with gFA11 and Mut1 siRNAs (Figure 1). The cells were transfected every 3-4 days for a total of four times over 12 days. Cells were kept in DMEM + 5 mM BHB after the first transfection. *, $p < 0.05$ by Student's t test. Error bars represent means ± 1SD. The averages shown were calculated on three biological replicates and the experiments shown are representative of at least two independent experiments. (**b**). Morphology of primary FRDA GM3665B fibroblast transfected with gFA11 siRNA or Mut1 siRNA, or not transfected. Arrows indicate senescent-appearing cells. (**c**). Flow-cytometric side scattering (SSC) of GM3665B cells infected with a gFA11-encoding or control-encoding (C3)

vector. ***, p < 0.005 by Student's t test.

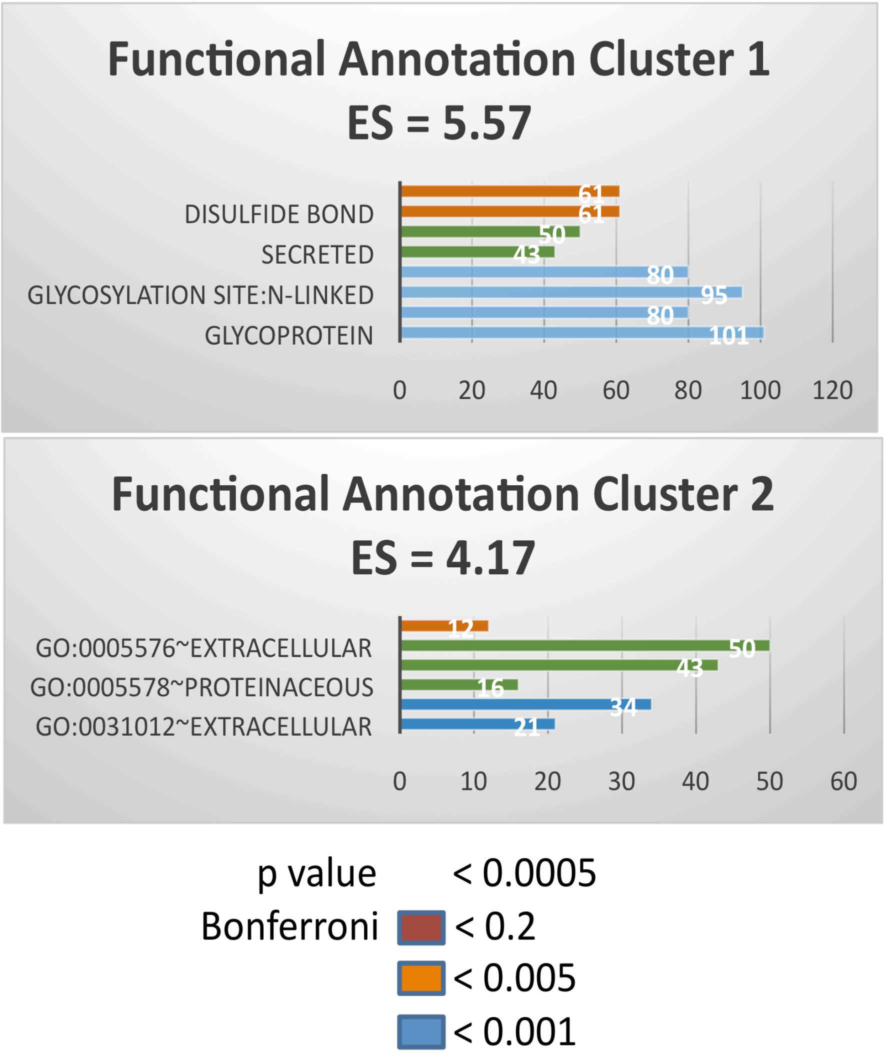

Figure S2. Clone gFA11 – *in silico* analysis. GM3816 FA fibroblasts were transfected with clone gFA11 siRNA or Mut1 siRNA for four times over twelve days in triplicate. After the first transfection the cells were grown in DMEM plus 5 mM BHB. After the fourth transfection, the RNA was extracted and used for a microarray analysis using the Affymetrix Human Gene 2.0 ST Array Gene chip. The top 301 genes (q=0) were used for a Functional Annotation Clustering analysis through D.A.V.I.D. v 6.7. Enrichment Score (ES) >1.3 are considered significant.

Fig.S3

a

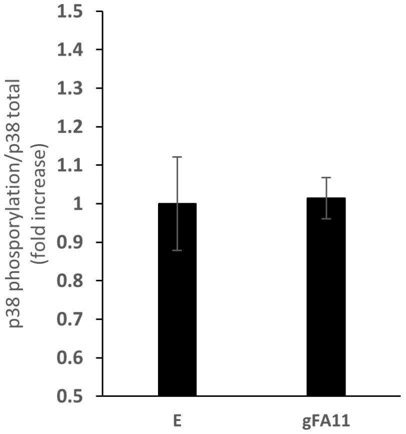

Figure S3. p38 phosphorylation is unaffected by gFA11. p38 phosphorylation status in apparently in GM3816 fibroblasts infected with an empty vector or with gFA11. The averages shown were calculated on four biological replicates. Error bars represent means ± 1SD.
